# Events in Early Life are Associated with Female Reproductive Ageing: A UK Biobank Study

**DOI:** 10.1038/srep24710

**Published:** 2016-04-20

**Authors:** Katherine S. Ruth, John R. B. Perry, William E. Henley, David Melzer, Michael N. Weedon, Anna Murray

**Affiliations:** 1 Genetics of Complex Traits, University of Exeter Medical School, RILD Level 3, Royal Devon & Exeter Hospital, Barrack Road, Exeter, EX2 5DW, UK; 2 MRC Epidemiology Unit, University of Cambridge School of Clinical Medicine, Box 285 Institute of Metabolic Science, Cambridge Biomedical Campus, Cambridge, CB2 0QQ, UK; 3 Health Statistics Group, University of Exeter Medical School, St Luke’s Campus, Exeter EX1 2LU, UK; 4 Epidemiology and Public Health, University of Exeter Medical School, Barrack Road, Exeter EX2 5DW, UK

## Abstract

The available oocyte pool is determined before birth, with the majority of oocytes lost before puberty. We hypothesised that events occurring before birth, in childhood or in adolescence (‘early-life risk factors’) could influence the size of the oocyte pool and thus the timing of menopause. We included cross-sectional data from 273,474 women from the UK Biobank, recruited in 2006–2010 from across the UK. We analysed the association of early menopause with events occurring before adulthood in 11,781 cases (menopause aged under 45) and 173,641 controls (menopause/pre-menopausal at ≥45 years), in models controlling for potential confounding variables. Being part of a multiple birth was strongly associated with early menopause (odds ratio = 1.42, confidence interval: 1.11, 1.82, *P* = 8.0 × 10^−9^, fully-adjusted model). Earlier age at menarche (odds ratio = 1.03, confidence interval: 1.01, 1.06, *P* = 2.5 × 10^−6^) and earlier year of birth were also associated with EM (odds ratio = 1.02, confidence interval: 1.00, 1.04, *P* = 8.0 × 10^−6^). We also confirmed previously reported associations with smoking, drinking alcohol, educational level and number of births. We identified an association between multiple births and early menopause, which connects events pre-birth, when the oocyte pool is formed, with reproductive ageing in later life.

Age at menopause influences health in later life with earlier menopause associated with increased osteoporosis and cardiovascular disease, and poorer cognitive function, but lower risks of several reproductive cancers^1^,^2^. Over half of the population variation in menopause age is estimated to be non-genetic, however only smoking and nulliparity have been reproducibly linked to earlier menopause^3^.

Natural menopause occurs on average at 51 years of age in Caucasian populations when the number of oocytes in the ovary are reduced below about 1000, however the factors that determine the timing of this event are poorly understood^4^. The number of ovarian follicles is determined before birth: approximately 7 million oocytes are produced by 6 months post conception, though this number declines rapidly before birth, continuing after birth so that by puberty only about 400,000 primary oocytes remain. Over 99% of ovarian follicle loss is due to atresia^5^, hence the rate of loss will influence age at menopause^3^. Menopause under the age of 45 affects approximately 5% of women and is of clinical relevance since it is associated with increased morbidity and mortality and affected individuals may benefit from treatment with hormone therapy^6^.

Early life events (defined in this study as occurring before birth, in childhood or in adolescence) have shown inconsistent relationships with age at menopause^3^. Previously reported positive associations with menopause include birth weight^7^,^8^,^9^, birth year^10^, whether breast fed as a baby^11^,^12^, food deprivation^13^,^14^, age at menarche^15^ childhood socio-economic status^16^, maternal smoking during pregnancy^17^, and weight in early childhood^7^,^11^,^12^. However, several studies have also reported null associations with these same traits, creating substantial uncertainty in the epidemiological literature^10^,^18^,^19^,^20^.

We hypothesised that pre-birth and early-life events could influence the oocyte pool and thus influence the timing of menopause. In this, one of the largest epidemiological analyses of age at menopause, we investigated whether early life variables are associated with the clinically-relevant outcome of menopause below the age of 45 years (early menopause (EM)). We investigated the early-life risk factors birth year, maternal smoking around birth, birth weight, being part of a multiple birth, whether breastfed as a baby, whether adopted as a child, handedness, comparative height and body size at age 10, age of menarche and illnesses occurring at under 20 years-of-age.

## Methods

### Source of data

We analysed data from 273,474 women from the UK Biobank, which includes 503,325 people aged 40–69 years recruited in 2006–2010 from across the UK^21^ (further details in Supplementary Methods).

### Identification of early menopause cases and controls

**Figure 1:**
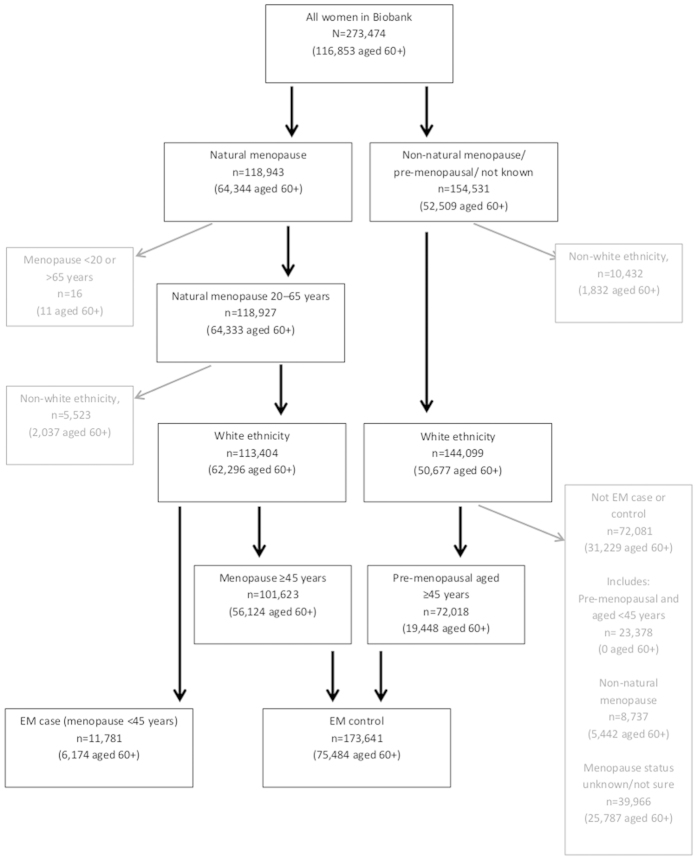
Selection of data used in the analysis.

We considered the outcome EM in our analyses, since this is clinically relevant and captures the lower extreme of normal reproductive ageing. EM cases were women with menopause under 45 years while controls had menopause or were known to be pre-menopausal at age 45 or over. Age at natural menopause was defined as age at last menstrual period excluding those with surgical menopause or taking hormone replacement therapy (further details in Supplementary Methods). Of the 257,503 white women, there were 11,781 EM cases (4.6%) and 173,641 controls (67.4%) (Fig. 1).

### Early life variables

We hypothesised that early-life risk factors influence the timing of menopause. In this study, we defined an early-life risk factor as a risk-factor occurring before adulthood, i.e. before birth, in childhood or in adolescence. Early-life risk factors have a clear temporal relationship with menopause and are suitable for analysis in cross-sectional data since they are unlikely to be biased by, or cause bias in, age at menopause. Sixteen early-life risk factors were present in the data: birth year, maternal smoking around birth, birth weight, part of a multiple birth, breastfed as a baby, adopted as a child, handedness, comparative height at age 10, comparative body size at age 10, age of menarche and six illnesses occurring at under 20 years-of-age. Illnesses diagnosed under the age of 20 were analysed in broad categories and were selected on the basis of frequency and relevance to reproductive phenotypes: ‘allergy’, ‘diabetes’, ‘infections’, ‘headaches’, ‘gynaecological issues’ and ‘cancer’ (more details are provided in Supplementary Methods). Women providing no answer to any of the relevant questions, answering ‘Prefer not to answer’ or answering ‘Not sure’ were coded as missing.

### Statistical methods

We used logistic regression to conduct analyses of early-life variables and odds of EM (*n* included from 98,273 to 181,778). All analyses were performed in Stata/SE v13.1. We restricted our analyses to women of white ethnicity (95% of cohort) and removed extreme outliers as appropriate (see Supplementary Methods). Potential confounding variables were controlled for in two ways: Firstly, the ‘partially-adjusted’ model included variables associated with age at menopause in published studies that were significant in exploratory univariate analyses of our UK Biobank data (approximately 1000 variables tested), and included Townsend deprivation index, BMI and smoking status; Secondly, the ‘fully-adjusted’ model, included all variables significantly associated with menopause in exploratory univariate analyses, and included smoking pack-years, frequency of alcohol intake, number of live births, educational level and whether the participant ate meat.

We included all available individuals including those with some missing data in each analysis. We also conducted sensitivity analyses in women with only complete data for all variables tested (*n* = 78,042): both sets of results were consistent. Due to the number of variables present in our UK Biobank data (approximately 1000), we used a conservative significance threshold of P < 5 × 10^−5^ for all tests and calculated 99.995% confidence intervals (CIs) around the odds ratio (OR).

### Consistency of relationships and sensitivity analyses

We performed parallel analysis using Cox proportional hazards regression models to check for consistency of relationships with menopause as a quantitative trait (Supplementary Methods) (*n* included from 126,001 to 228,221). To investigate the sensitivity of our results to age at recruitment which was different in cases and controls (Supplementary Fig. 1), we performed the same analyses in a subset of women aged 60 and over. Of 112,887 women aged 60 and over, there were 6,174 EM cases (5.5%) and 75,484 controls (66.9%) (Fig. 1). We investigated whether results that were significant for EM were also significant for primary ovarian insufficiency (POI) by conducting logistic regression in 2,549 POI cases (menopause under 40 years) and 218,269 controls (known to be pre-menopausal at age 40 or over).

## Results

### Distribution of age at menopause is skewed

We identified 113,417 women with natural menopause, with a mean age at menopause of 50 years (Table 1). The distribution of age at menopause was skewed even when only women aged 60 and over were considered, and had peaks at values ending in zero, two and five (Supplementary Fig. 2). Descriptive statistics for the cohort are presented in Table 2 and Supplementary Table 1.

### Associations of potential confounding variables

**Figure 2:**
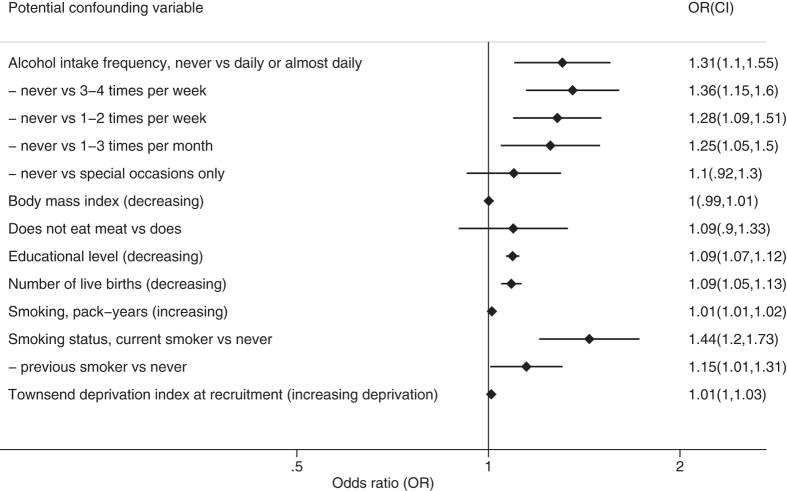
Associations of potential confounding variables with early menopause. Results shown are for the fully-adjusted logistic regression model including the potential confounding variables Townsend deprivation index, BMI, smoking status, smoking pack-years, frequency of alcohol intake, number of live births, educational level and whether the participant ate meat (*n* = 152,701). Confidence intervals are 99.995%.

Being a current smoker had the largest effect on EM (for current smokers compared with never smokers, OR = 1.44, CI: 1.20, 1.73, *P* < 1 × 10^−15^) (Fig. 2) (Supplementary Tables 2 and 3). Other non-early life factors associated with earlier menopause were never drinking alcohol (e.g. OR = 1.28, CI: 1.09, 1.51, *P* = 2.5 × 10^−10^ for never drinking compared with drinking 1–2 times per week), decreasing educational level (OR = 1.09, CI: 1.07, 1.12, *P* < 1 × 10^−15^ per level), and fewer live births (OR = 1.09, CI: 1.05, 1.13, *P* < 1 × 10^−15^ per birth).

### Events before birth are associated with EM

**Figure 3:**
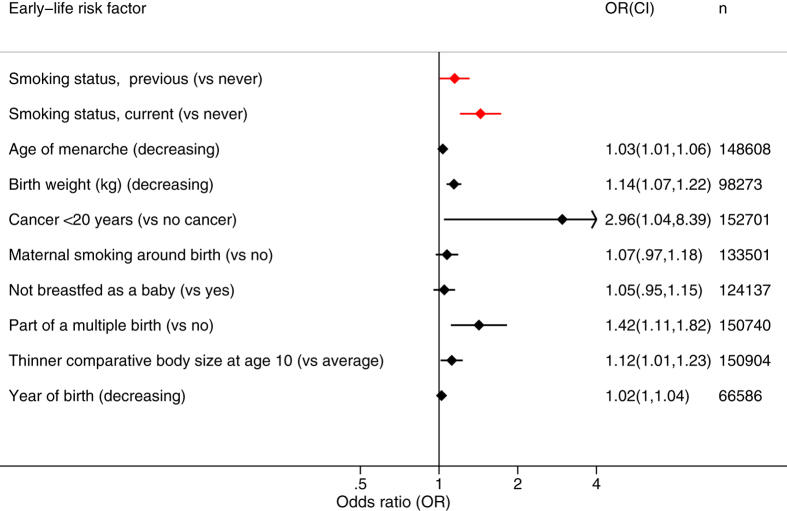
Associations between early-life risk factors and early menopause. Results are for the logistic regression model including one early-life risk factor at a time, adjusted for the potential confounding variables Townsend deprivation index, BMI, smoking status, smoking pack-years, frequency of alcohol intake, number of live births, educational level and whether the participant ate meat. Analyses are in all ages, except for year of birth which was analysed in women aged 60 and over at recruitment. Smoking status is included for reference. Confidence intervals are 99.995%. To achieve the same effect size as being a current smoker (the binary variable with nominally the largest effect size), for the continuous variables the required change in risk factor would be: age at menarche, 11.1 years (7.0 s.d.); birth weight 2.8 kg (4.7 s.d.); year of birth, 16.9 years (2.1 s.d.).

Earlier year of birth was associated with EM (OR = 1.02, CI: 1.00, 1.04, *P* = 8 × 10^−6^) in the partially- and fully-adjusted models, as was decreasing birth weight and being part of a multiple birth (Fig. 3) (Supplementary Tables 2 and 3). For the effect of year of birth on EM to be as large as being a current smoker, a women would need to be born 16.9 years earlier (2.1 standard deviations).

Maternal smoking was significantly associated with EM only in the partially-adjusted model. The effect size for being part of a multiple birth (OR = 1.42, CI: 1.11, 1.82, *P* = 8 × 10^−9^, fully-adjusted model) was similar to that of being a current smoker (OR = 1.44, CI: 1.20, 1.73, *P* < 1 × 10^−15^, fully-adjusted model). These associations remained when multiple birth, decreasing birth weight and maternal smoking were considered in the same logistic regression model (Supplementary Table 4).

Since the association of EM with birth weight is adjusted for BMI, it could be considered that this is actually an association between EM and change in size^22^. The association with birth weight persisted regardless of adjustment for BMI (fully-adjusted model including BMI, OR = 1.14 per kg, *P* = 2.5 × 10^−10^; fully-adjusted model without BMI, OR = 1.14 per kg, *P* = 2.3 × 10^−10^), indicating that the association was with birth weight and not change in size.

### Events in childhood and adolescence are associated with earlier menopause

Earlier age at menarche was associated with EM in the fully-adjusted logistic model (OR = 1.03, CI: 1.01, 1.06, *P* = 2.5 × 10^−6^), but not the partially-adjusted model (Fig. 3, Supplementary Table 2). The perception of being ‘thinner at age 10’ was significantly associated with EM in the partially- and fully-adjusted models (OR = 1.12, CI: 1.01, 1.23, *P* = 3.5 × 10^−6^ for fully-adjusted) (Fig. 3). When comparative body size at age 10 was included with age at menarche in the model (Supplementary Table 5), both remained significantly associated with EM. The description of being ‘plumper at age 10’ was not associated with age at menopause in any of the models. Handedness, whether adopted as a child and being breastfed were not associated with EM (Fig. 3) (Supplementary Tables 2 and 3).

### EM is associated with cancer but not with other illnesses

Considering illnesses under the age of 20, only cancer was associated with EM (Fig. 3, Supplementary Tables 2 and 3), but only in the fully-adjusted model (OR = 2.96, CI: 1.04, 8.39, *P* = 2.4 × 10^−5^). There were no significant associations with allergy, gynaecological problems, infections and headache/migraine.

### Consistency of relationships in Cox proportional hazards model of menopause as a quantitative trait and sensitivity analysis

Using Cox proportional hazards regression, we were able to explore whether associations with EM also held for menopause as a quantitative trait, where the outcome is hazard ratio (HR) of menopause. The potential confounding variables that were significantly associated with EM – drinking alcohol, educational level, number of live births, and smoking – also had consistent directions of effect in the Cox model. Increasing social deprivation, lower BMI and not eating meat were associated with increased HR of menopause, though these risk factors were not associated with EM (Supplementary Tables 2 and 3) (Supplementary Fig. 3). The directions of effect for the early-life risk factors were consistent in the EM and Cox models (Supplementary Tables 2 and 3). In the analysis based on women aged 60 and over, the direction of effects was consistent with the analysis in all age groups (Supplementary Table 3). In the analysis of the outcome POI, none of the early-life variables reached our significance threshold though directions of effect were consistent (Supplementary Table 6).

### Multiple birth and menarche remain strongly associated in models including all early-life risk factors

**Figure 4:**
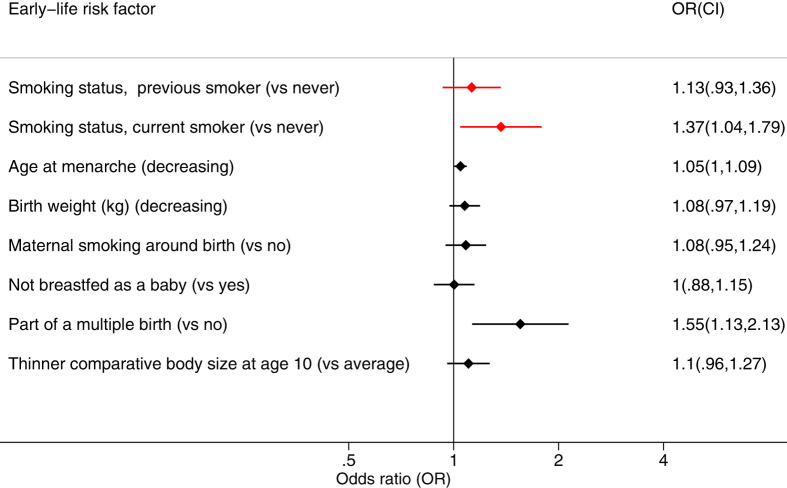
Associations in the multiple early-life risk factor model. Results shown are for the logistic regression model in all age groups including all early-life risk factors at the same time (*n* = 78,603), adjusted for the potential confounding variables Townsend deprivation index, BMI, smoking status, smoking pack-years, frequency of alcohol intake, number of live births, educational level and whether the participant ate meat. The early life risk-factors included are age at menarche, birth weight, maternal smoking, breast fed as a baby, part of multiple birth and comparative body size at age 10. Cancer at under 20 years and year of birth were not included due to the small number of cases in the former, and confounding with age at recruitment in the latter. Confidence intervals are 99.995%.

In a fully-adjusted model including all early-life risk factors that were associated with EM (age at menarche, birth weight, maternal smoking, breast fed as a baby, part of multiple birth and comparative body size at age 10), only the risk factors younger age at menarche (OR = 1.05, CI: 1.0, 1.09, *P* = 7.27 × 10^−6^) and being part of a multiple birth (OR = 1.55, CI: 1.13, 2.13, *P* = 2.11 × 10^−8^) remained significantly associated with EM (Fig. 4, Supplementary Table 7). Only age at menarche remained significantly associated in the Cox model including all early-life risk factors (Supplementary Table 7). Cancer at under 20 years and year of birth were not included in the model including all early-life risk factors, due to the small number of cases in the former, and bias due to age at recruitment in the latter.

## Discussion

Our study is the largest to date on the effect of non-genetic risk factors on age at menopause, compared with the previous largest studies in 95,704 US women and 50,678 UK women^15^,^23^. In this study, we have shown that pre-birth and early-life risk factors are associated with EM, an event in later life. Being part of a multiple birth demonstrated the most robust association, with an effect size comparable to being a smoker in this study, the single biggest predictor previously identified^10^,^15^,^18^,^19^,^23^,^24^,^25^. The association with multiple births persisted when we included all significant early-life variables and adjusted for all potential confounders. Non-early life risk factors are better characterised in the current literature than early-life variables and our results were consistent with previous studies. We found that odds of EM were increased by being a current smoker^10^,^15^,^18^,^19^,^23^,^24^,^25^, having decreased levels of education^18^,^19^,^25^,^26^ and being nulliparous^15^,^23^. However, we did not find consistent associations between age at menopause and BMI^15^,^23^, socio-economic status and not eating meat^16,23,27^, as suggested by previous studies.

Being part of a multiple birth and birth weight were the pre-birth risk factors with the strongest associations with EM. An association of EM with multiple births has not previously been reported, though the prevalence of POI has been found to be higher in monozygotic and dizygotic twins^20^,^28^. This suggests that the shared intra-uterine or early postnatal environment may play a role, or that there could be overlap in the genetics of twinning and age at menopause. The rate of multiple births in our study (2.4%) was slightly lower than recent estimates (3%), since the rate has increased in recent years due to assisted reproduction^9^,^29^,^30^. The association of multiple births with EM in this study should be independent of any potential effects of assisted reproduction since women in the study were born prior to its introduction. Babies from a multiple pregnancy are more likely to suffer complications including intrauterine growth restriction and are smaller at birth. However, intra-uterine growth restriction has not been found to restrict ovary growth and development^31^. Multiple births are more common as women age^30^ and therefore the size of the oocyte pool could be reduced in offspring of older mothers. This could be mediated by genetic abnormalities, hypertension or birth complications, which are known to be more common in older women. However, we tested for effects of increased maternal age in our data and found no increased odds of EM, in fact there was a modest effect in the opposite direction, though we were unable to adjust for potential confounding factors such as maternal socio-economic status and age at menopause and survivor bias. In our study there was an association of EM with birth weight, however the effect was less strong than that of multiple births but this may be because we were unable to adjust our analysis by gestational age. Evidence from other studies of the effects of birth weight has been contradictory^7^,^9^,^20^,^32^.

The association between earlier age at menarche and increased odds of EM agrees with another analysis using the UK Biobank data^33^, and is supported by findings from a study on over 90,000 women^15^ and a smaller study on several thousand women^34^ which both found associations between earlier menarche and earlier menopause. In addition, a published study has reported a correlation (using LD score regression) between genetic variants associated with age at menarche and age at menopause (r_g_ = 0.14, *P* = 0.003) and five loci associated with age at menopause contain genes associated with hypogonadotropic hypogonadism^50^. We found that being thinner at age 10 was associated with increased odds of EM. It is unlikely that this association is driven by an effect on menarche timing as being thinner in childhood is associated with delayed menarche^35^,^36^ and in our data early menarche was a risk factor for EM. The role of childhood nutrition has been highlighted by previous studies that found associations between not being breast fed and earlier age at menopause^11^,^12^ (though this was not significantly associated with EM in our study) and earlier menopause in women who are lighter at 1–2 years^7^,^11^,^12^ or who have been exposed to famine in early childhood^37^.

In our cohort of women born in 1936–1970, there was an increased odds of EM in women born in earlier birth years. We estimate an increase in age at menopause of about 1.3–2.5 months per year of later birth (data not presented), which agrees with two smaller studies on cohorts born between 1908–1930 in Sweden (n = 1,017) and 1912–1969 in the USA (n = 22,851)^34^,^38^. The US study found a 17-month increase in age at menopause during 1915–1939^38^, while the Swedish study found an increase of 1.2 months per later birth year.

In our analysis of illnesses occurring before the age of 20, only cancer showed an association with EM. It is likely that cancer treatment will have had an effect on age at menopause – such treatment is known to affect fertility^39^ and has late side-effects such as cardiovascular disease and cancers in later life^40^,^41^. The illness data used were self-reported and may possibly be affected by recall bias^42^,^43^. By restricting these analyses to cases under 20 we have minimised uncertainty about cause and effect of the illness and menopause. However, compared to a prospective study of incident cases, our cohort will be biased since the women included will have had to have survived until the study

A limitation of this study is that the UK Biobank data is based on volunteers and so there may be selection bias. The data used in our analysis was collected retrospectively and so may be subject to recall bias. Clear rounding of menopause age was evident, as has been seen in previous studies^44^. Previous studies have indicated that menopause is reasonably well recalled, though error increases with time after menopause^45^,^46^. Ethnicity has been shown to be associated with age at menopause^15^,^18^,^26^ and we restricted our analysis to white women as we had insufficient numbers of women of other ethnicities to conduct sub-analyses. Age at menopause is limited by age at recruitment, however this should not confound the effect of variables other than year of birth, which was analysed in a post-menopausal cohort to address this. In addition there could be additional confounders that were not captured by our data. There was variation in the number of responses for each variable, with only about 40% of the case–control cohort included in the full analysis model with all early-life risk factors, and we cannot rule out that such missing-ness could be informative.

Due to the number of women included in this study, we provide powerful evidence for the influence of early-life risk factors on EM, and hence women’s health. More specifically, that being a multiple birth, having menarche at a younger age, and being born in an earlier birth cohort are associated with EM. It is plausible that early-life events could influence the oocyte pool since the number of ovarian follicles is determined before birth and the number of primary oocytes decline dramatically prior to puberty – from about 7 million oocytes at 6 months post conception to about 400,000 oocytes by puberty. Early life events identified in our study could affect the oocyte reserve *in utero* or in early development and could be mediated through increased stress to oocytes, perhaps caused by reduced nutritional or oxygen availability. In rats, suboptimal nutrition during pregnancy is associated with transgenerational accelerated female reproductive ageing^47^. Factors affecting the number of follicles and their quality, and the rate of follicle decline both in utero and following birth could plausibly affect age at menopause, which itself is a consequence of a reduction in the follicle pool. At the cellular level, DNA repair has been identified as an important mechanism influencing age at menopause and may result in maintenance of a larger, better quality follicle pool^48^,^49^. Since up to 50% of population variation in age at menopause is thought to be due to genetics, risk factors affecting the size of the follicle pool are likely to have different effects on age at menopause according to the genetic variants present. We anticipate that these findings should provide direction for further work into the biological processes driving menopause, and in particular how genetics and the environment interact.

## Additional Information

**How to cite this article**: Ruth, K. S. *et al.* Events in Early Life are Associated with Female Reproductive Ageing: A UK Biobank Study. *Sci. Rep.* **6**, 24710; doi: 10.1038/srep24710 (2016).

## Supplementary Material

Supplementary Information

Supplementary Dataset

## Figures and Tables

**Table 1:** Age at natural menopause of women with self-reported white ethnicity in UK Biobank.

	*n*	*N*	%	Mean age at menopause in years (standard deviation)	Median age at menopause in years (range)
Natural menopause	113,417	257,516	44.0	50.0 (4.5)	50 (18,65)
Early menopause (<45 years)	11,794	233,394	5.1	40.6 (3.5)	42 (18,44)

*n* is number of women with natural menopause or early menopause (includes women with menopause at <20 years or >65 years) and women with early menopause aged <45 years at recruitment). *N* is the total number of women for whom whom menopause (all women) or early menopause status (women aged 45 and over at recruitment) could be determined

**Table 2:** Descriptive statistics for early life variables.

		All cohort		Natural menopause		Early menopause (<45 years)	
Age of menarche	n	250,143		111,034		11,565	
	mean	13.0		13.0		12.9	
	s.d.	1.6		1.6		1.7	
	median (range)	13 (5,25)		13 (5,25)		13 (5,21)	
Birth weight (kg)	n	164,448		69,367		7,210	
	mean	3.2		3.2		3.2	
	s.d.	0.6		0.6		0.7	
	median (range)	3.2 (0.4,9)		3.2 (0.4,9)		3.2 (0.7,6.3)	
Year of birth	n	257,516		113,417		11,794	
	mean	1951.5		1948.4		1949.6	
	s.d.	8.0		5.6		7.1	
	median (range)	1950 (1936,1970)		1948 (1936,1969)		1948 (1936,1969)	
		**n**	**%**	**n**	**%**	**n**	**%**
Breastfed as a baby	No	64,388	31.0	23,563	26.1	2,807	30.1
	Yes	143,195	69.0	66,689	73.9	6,531	69.9
	Total	207,583		90,252		9,338	
Comparative height at age 10	Shorter	53,619	21.2	23,549	21.1	2,502	21.6
	Taller	64,628	25.5	28,113	25.2	2,987	25.8
	Average	134,773	53.3	59,943	53.7	6,105	52.7
	Total	253,020		111,605		11,594	
Comparative body size at age 10	Thinner	79,326	31.3	34,402	30.7	3,890	33.5
	Plumper	45,203	17.8	19,358	17.3	2,050	17.6
	Average	129,271	50.9	58,170	52.0	5,685	48.9
	Total	253,800		111,930		11,625	
Maternal smoking around birth	No	156,728	70.3	70,228	71.6	6,977	68.2
	Yes	66,291	29.7	27,904	28.4	3,256	31.8
	Total	223,019		98,132		10,233	
Part of a multiple birth	No	247,777	97.7	109,292	97.6	11,242	96.9
	Yes	5,928	2.3	2,637	2.4	365	3.1
	Total	253,705		111,929		11,607	

Based on women with self-reported white ethnicity in UK Biobank. Women providing no answer, answering ‘Prefer not to answer’ or answering ‘Not sure’ were excluded from the analysis.
