# Supplementary material for: Events in Early Life are Associated with Female Reproductive Ageing: A UK Biobank Study
Source: Sci Rep. 2016 Apr 20;6:24710. doi: 10.1038/srep24710 (PMC4837365; doi:10.1038/srep24710)
Supplement: Supplementary Information [file srep24710-s1.pdf]

Supplementary Information 1

Events in Early Life are Associated with Female Reproductive Ageing: A UK Biobank Study

Katherine S Ruth[1], John RB Perry[2], William E Henley[3], David Melzer[4], Michael N Weedon[1], Anna Murray[*1]

[1]Genetics of Complex Traits, University of Exeter Medical School, RILD Level 3, Royal Devon & Exeter Hospital, Barrack Road, Exeter, EX2 5DW
[2]MRC Epidemiology Unit, University of Cambridge School of Clinical Medicine, Box 285 Institute of Metabolic Science, Cambridge Biomedical Campus, Cambridge, CB2 0QQ, UK
[3]Health Statistics Group, University of Exeter Medical School, St Luke's Campus, Exeter EX1 2LU, UK
[4]Epidemiology and Public Health, University of Exeter Medical School, Barrack Road, Exeter EX2 5DW, UK

Correspondence to: Dr Anna Murray (a.murray@exeter.ac.uk)

Contents

Supplementary Methods

Supplementary Results

Supplementary Figure 1. Distribution of age at recruitment in early menopause cases and controls.

Supplementary Figure 2. Distribution of age at menopause in all women and women aged 60 and over at recruitment.

Supplementary Methods

Source of data

UK Biobank invited 9.2 million people to participate, giving a response rate of 5.47% [1]. Participants were registered with the NHS and lived within 25 miles of one of the 22 assessment centres. Participants answered detailed questions about themselves, had measurements taken and provided samples. The data included follow-up data collected in 2012–2013 for 20,000 participants from North West England. In our analysis we analysed baseline data for 266,237 people with a single visit, and follow-up data for 7,237 participants.

Age at menopause

A total of 166,659 women stated that they were menopausal, and of these 155,658 (93%) gave an age at menopause; of the rest, 10,485 (6%) did not know, 378 (0.2%) preferred not to answer, and 48 (0.03%) had missing values. Of the women with an age at menopause, 9,154 (5.9%) were excluded as they were missing information relating to whether they had had hysterectomy, oophorectomy or HRT, or the age at which this took place. A total of 10,779 women with an age at menopause were excluded as they had menopause due to hysterectomy or oophorectomy (6.9%), with a further 16,782 (10.8%) excluded as HRT was ongoing at time of menopause. Overall we identified 118,943 women with an age at natural menopause. We then restricted our analysis to women with a natural menopause between 20 and 65 years and to women of white ethnicity, giving 113,404 women with natural menopause who were identified.

Exclusion of outliers

Women with menopause under 20 or over 65 were excluded (n=16) on the basis of biological plausibility. Age at menarche was restricted to a clinically normal range of 9–17 years. Birth weights in excess of 6kg were excluded (n=86) as implausible/extreme outliers.

Cox proportional hazards model

Cox proportional hazards regression was run using the stset and stcox commands in Stata using age as the time variable, starting at birth (0 years) and ending at last age at risk of natural menopause, with natural menopause as the event. Women included in the analysis were censored at bilateral oophorectomy and/or hysterectomy, or start of HRT use (where this was ongoing at time of menopause, hysterectomy or oophorectomy). Covariates were considered to be constant throughout the time at risk. The Cox proportional hazards assumption was tested using Schoenfeld residuals, however in most cases this did not hold.

Variables included

For further details of the variables included, please see http://www.ukbiobank.ac.uk/

Questions used to define menopause.

Age at menopause was derived from the following. Women providing no answer to any of the relevant questions, answering ‘Prefer not to answer’ or answering ‘Not sure’ were excluded from the analysis.

2724: Had menopause. Have you had your menopause (periods stopped)?

- Prefer not to answer
- No
- Yes
- Not sure - had a hysterectomy
- Not sure - other reason

3581: Age at menopause. How old were you when your periods stopped?

3591: Ever had hysterectomy?

- Not sure
- Prefer not to answer
- No
- Yes

2824: Age at hysterectomy?

2834: Bilateral oophorectomy?

Not sure

Prefer not to answer

No

Yes

3882: Age at bilateral oophorectomy?

2814: Ever used HRT?

Prefer not to answer

Do not know

No

Yes

3536: Age started HRT?

3546: Age last used HRT?

Questions used to define the early life variables

Women providing no answer to any of the relevant questions, answering ‘Prefer not to answer’ or answering ‘Not sure’ were excluded from the analysis.

34: Year of birth

1787: Maternal smoking around birth

Prefer not to answer

Do not know

No

Yes

20022: Birth weight

1777: Part of a multiple birth

Prefer not to answer

Do not know

No

Yes

1677: Breastfed as a baby

- Prefer not to answer
- Do not know
- No
- Yes

1767: Adopted as a child

- Prefer not to answer
- Do not know
- No
- Yes

1707: Handedness (chirality/laterality)

- Prefer not to answer
- Right-handed
- Left-handed
- Use both right and left hands equally

1697: Comparative height at age 10

- Prefer not to answer
- Do not know
- Shorter
- Taller
- About average

1687: Comparative body size at age 10

- Prefer not to answer
- Do not know
- Thinner
- Plumper
- About average

2714: Age when periods started (menarche)

Questions used to define the potential confounding variables

Women providing no answer to any of the relevant questions, answering ‘Prefer not to answer’ or answering ‘Not sure’ were excluded from the analysis.

189: Townsend deprivation index at recruitment

1558: Alcohol intake frequency

- Prefer not to answer
- Daily or almost daily
- Three or four times a week
- Once or twice a week
- One to three times a month
- Special occasions only
- Never

2734: Number of live births

20116: Smoking status

- Prefer not to answer
- Never
- Previous
- Current

21001: Body mass index (BMI)

Educational level was derived from:

- 6138: Qualifications
    - None of the above
    - Prefer not to answer
    - College or University degree
    - A levels/AS levels or equivalent
    - O levels/GCSEs or equivalent
    - CSEs or equivalent
    - NVQ or HND or HNC or equivalent
    - Other professional qualifications eg: nursing, teaching

Pack-years was derived using:

- 20116: Smoking status
  - Prefer not to answer
  - Never
  - Previous
  - Current
- 2867: Age started smoking in former smokers
- 2887: Number of cigarettes previously smoked daily
- 2897: Age stopped smoking
- 3436: Age started smoking in current smokers
- 3456: Number of cigarettes currently smoked daily (current cigarette smokers)
- 21003: Age at recruitment

Whether the participant ate meat was derived from:

- 1359: Poultry intake
  - Prefer not to answer
  - Do not know
  - Never
  - Less than once a week
  - Once a week
  - 2-4 times a week
  - 5-6 times a week
  - Once or more daily
- 1369: Beef intake
  - Prefer not to answer
  - Do not know
  - Never
  - Less than once a week
  - Once a week
  - 2-4 times a week
  - 5-6 times a week
  - Once or more daily

1379: Lamb/mutton intake

- Prefer not to answer
- Do not know
- Never
- Less than once a week
- Once a week
- 2-4 times a week
- 5-6 times a week
- Once or more daily

1389: Pork intake

- Prefer not to answer
- Do not know
- Never
- Less than once a week
- Once a week
- 2-4 times a week
- 5-6 times a week
- Once or more daily

Illnesses included (all have at least one case at under 20 years)

Cancer aged under 20 was identified from ‘20007: Interpolated age of participant when cancer first diagnosed’. Other illnesses were identified from ‘20002: Non-cancer illness code, self-reported’ and ‘20009: Interpolated age of participant when non-cancer illness first diagnosed’.

Allergy<br>N=16,779	Cancer<br>N=241	Diabetes<br>N=332
Allergy or anaphylactic reaction to drug	Any self-reported cancer reported under 20 years:	T1 diabetes
Allergy or anaphylactic reaction to food	Acute myeloid leukaemia	Diabetes
Allergy to house dust mite	Basal cell carcinoma	
Allergy/ hypersensitivity/ anaphylaxis	Bladder cancer	
Asthma	Brain cancer / primary malignant brain tumour	
Blistering/ desquamating skin disorder	Breast cancer	
Contact dermatitis	Cancer of lip/ mouth/ pharynx/ oral cavity	
Crohn’s disease	Cervical cancer	
Eczema/ dermatitis	Chronic lymphocytic	
Food intolerance	Chronic myeloid	
Hay fever/ allergic rhinitis	CIN/ pre-cancer cells cervix	
Psoriasis	Colon cancer/ sigmoid cancer	
Ulcerative colitis	Ear cancer	
	Eye and/ or adnexal cancer	
	Female genital tract cancer	
	Hodgkin’s lymphoma / Hodgkin’s disease	
	Kidney/ renal cell cancer	
	Large bowel cancer/ colorectal cancer	
	Leukaemia	
	Liver/ hepatocellular cancer	
	Lung cancer	
	Malignant lymph node, unspecified	
	Malignant melanoma	
	Meningeal cancer / malignant meningioma	
	Non-Hodgkin’s lymphoma	
	Non-melanoma skin cancer	
	Ovarian cancer	
	Parotid gland cancer	
	Peripheral nerve/ autonomic nerve cancer	
	Primary bone cancer	
	Retinoblastoma	
	Sarcoma/ fibrosarcoma	
	Skin cancer	
	Small intestine/ small bowel cancer	
	Spinal cord or cranial nerve cancer	
	Thyroid cancer	
	Unclassifiable	
	Uterine/ endometrial cancer	
	Vaginal cancer	

Continued on next page.

Gynaecological problems<br>N=660	Infections<br>N=10,164	Migraine/ headache<br>N=4,667
Abnormal smear (cervix)<br>Cervical erosion<br>Cervical polyps<br>Cervical problem<br>Dysmenorrhoea / dysmenorrhea<br>Ectopic pregnancy<br>Female infertility<br>Fibrocystic disease<br>Gynaecological disorder (not cancer)<br>Menopausal symptoms / menopause<br>Menorrhagia (unknown cause)<br>Miscarriage<br>Ovarian cyst or cysts<br>Ovarian problem<br>Pelvic inflammatory disease/PID<br>Polycystic ovaries/ polycystic ovarian syndrome<br>Uterine fibroids<br>Uterine polyps<br>Vaginal prolapse/ uterine prolapse	Acute infective polyneuritis/ Guillain-Barre syndrome<br>Appendicitis<br>Blepharitis/ eyelid infection<br>Bronchitis<br>Cellulitis<br>Chickenpox<br>Dengue fever<br>Diphtheria<br>Encephalitis<br>Gastroenteritis/dysentery<br>Helicobacter pylori<br>Hepatitis<br>Hepatitis A<br>Hepatitis B<br>Hepatitis C<br>Herpes simplex<br>Infection of nervous system<br>Infectious mononucleosis/ glandular fever/ Epstein Barr virus (EBV)<br>Infective/ viral hepatitis<br>Lung abscess<br>Malaria<br>Measles/ morbillivirus<br>Meningitis<br>Mumps/ epidemic parotitis<br>Non-infective hepatitis<br>Pericarditis<br>Peritonitis<br>Pneumonia<br>Polio/ poliomyelitis<br>Rheumatic fever<br>Rubella/ German measles<br>Scarlet fever/ scarlatina<br>Schistosomiasis/ bilharzia<br>Septicaemia/ sepsis<br>Shingles<br>Tonsillitis<br>Tuberculosis (TB)<br>Typhoid fever<br>Whooping cough/ pertussis<br>Yellow fever	Migraine<br>Headaches (not migraine)

N is number of women of white ethnicity recorded with illness. Note: each person can have more than one illness.

References (Supplementary Methods)

1 Allen, N. *et al.* UK Biobank: Current status and what it means for epidemiology. *Health Policy and Technology* **1**, 123-126, doi:http://dx.doi.org/10.1016/j.hlpt.2012.07.003 (2012).

Supplementary Results

Supplementary Figure 1. Distribution of age at recruitment in early menopause cases and controls.

(i) All cohort.

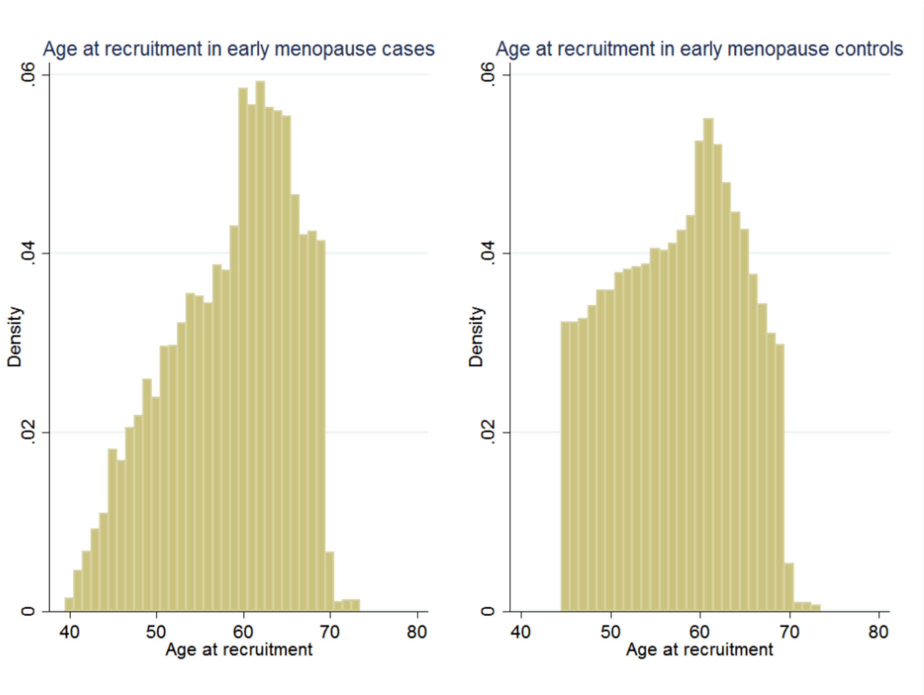

(ii) Aged 60 and over at recruitment.

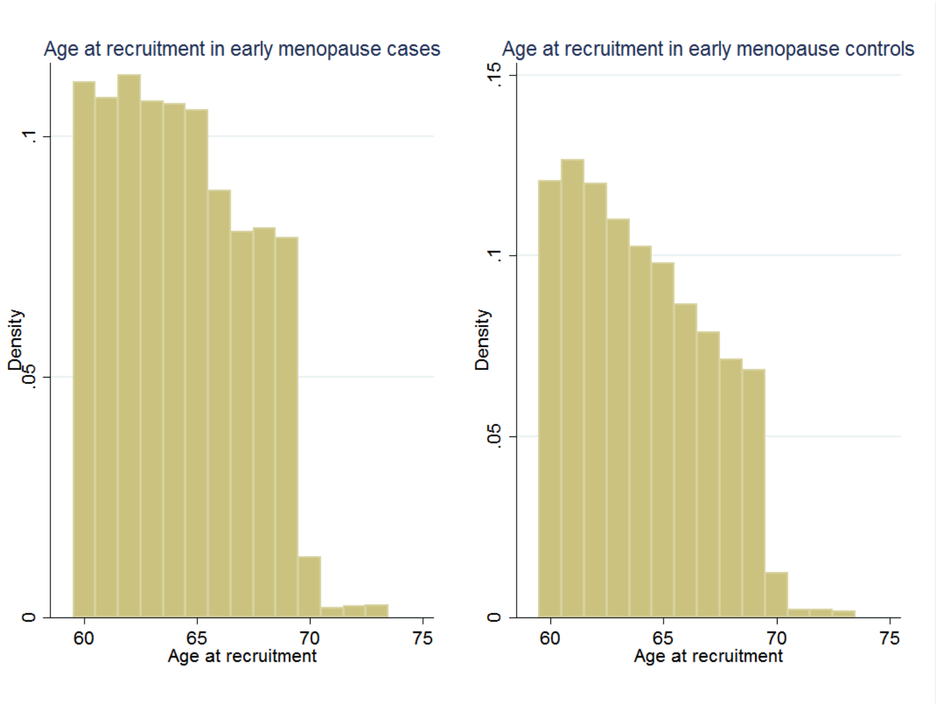

Supplementary Figure 2. Distribution of age at menopause in all women and women aged 60 and over at recruitment.

(i) All cohort.

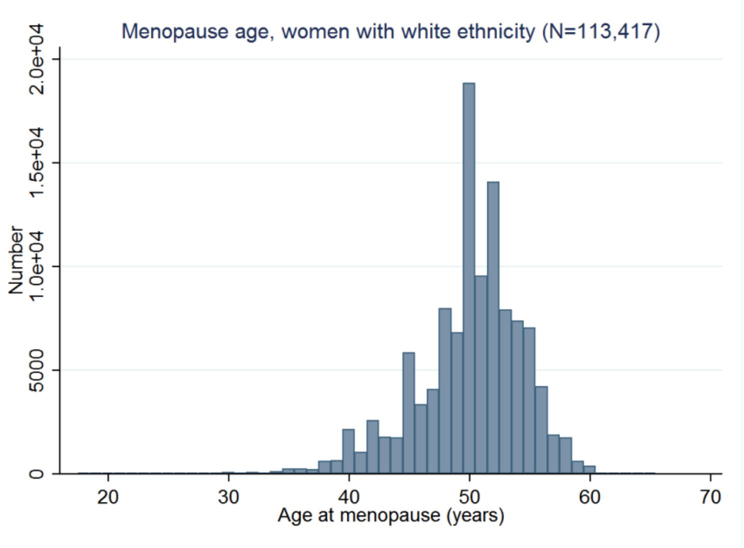

Median=50 years. Interquartile range (48,53). Mean=50.0 years. Range (18,65).

(ii) Aged 60 and over at recruitment.

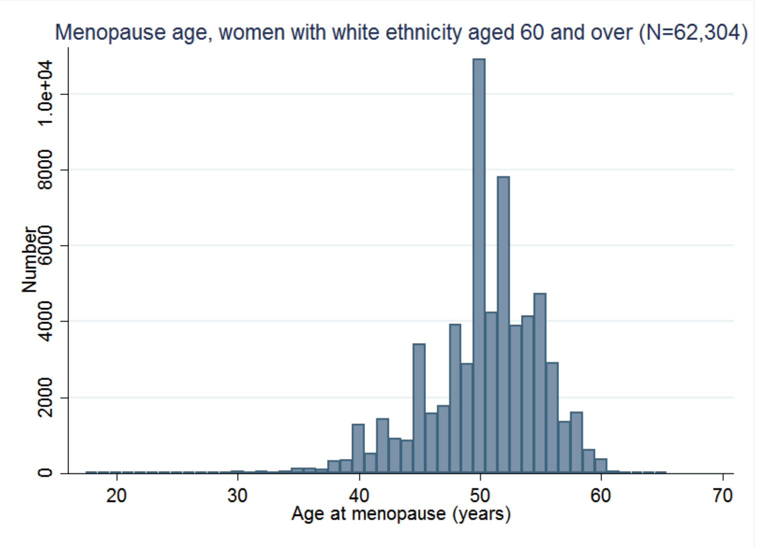

Median=51 years. Interquartile range (48,54). Mean=50.4 years. Range (18,65).
